# Bacterial Diversity on Historical Audio-Visual Materials and in the Atmosphere of Czech Depositories

**DOI:** 10.1128/spectrum.01176-23

**Published:** 2023-07-10

**Authors:** Tereza Branysova, Ondrej Limpouch, Michal Durovic, Katerina Demnerova, Hana Stiborova

**Affiliations:** a Faculty of Food and Biochemical Technology, Department of Biochemistry and Microbiology, University of Chemistry and Technology, Prague, Prague, Czech Republic; b Faculty of Chemical Technology, Department of Chemical Technology of Monument Conservation, University of Chemistry and Technology, Prague, Prague, Czech Republic; University of Minnesota Twin Cities

**Keywords:** air contamination, audio-visual materials, bacterial contamination, cultural heritage, Illumina MiSeq

## Abstract

Microbial contamination in cultural heritage storage facilities is undoubtedly still a huge problem and leads to the biodeterioration of historical objects and thus the loss of information for future generations. Most studies focus on fungi that colonize materials, which are the primary agents of biodeterioration. However, bacteria also play crucial roles in this process. Therefore, this study focuses on identifying bacteria that colonize audio-visual materials and those present in the air in the archives of the Czech Republic. For our purposes, the Illumina MiSeq amplicon sequencing method was used. Using this method, 18 bacterial genera with an abundance of higher than 1% were identified on audio-visual materials and in the air. We also evaluated some factors that were assumed to possibly influence the composition of bacterial communities on audio-visual materials, of which locality was shown to be significant. Locality also explained most of the variability in bacterial community structure. Furthermore, an association between genera colonizing materials and genera present in the air was demonstrated, and indicator genera were evaluated for each locality.

**IMPORTANCE** The existing literature on microbial contamination of audio-visual materials has predominantly used culture-based methods to evaluate contamination and has overlooked the potential impact of environmental factors and material composition on microbial communities. Furthermore, previous studies have mainly focused on contamination by microscopic fungi, neglecting other potentially harmful microorganisms. To address these gaps in knowledge, our study is the first to provide a comprehensive analysis of bacterial communities present on historical audio-visual materials. Our statistical analyses demonstrate the critical importance of including air analysis in such studies, as airborne microorganisms can significantly contribute to the contamination of these materials. The insights gained from this study are not only valuable in developing effective preventive measures to mitigate contamination but also valuable in identifying targeted disinfection methods for specific types of microorganisms. Overall, our findings highlight the need for a more holistic approach to understanding microbial contamination in cultural heritage materials.

## INTRODUCTION

**FIG 1:**
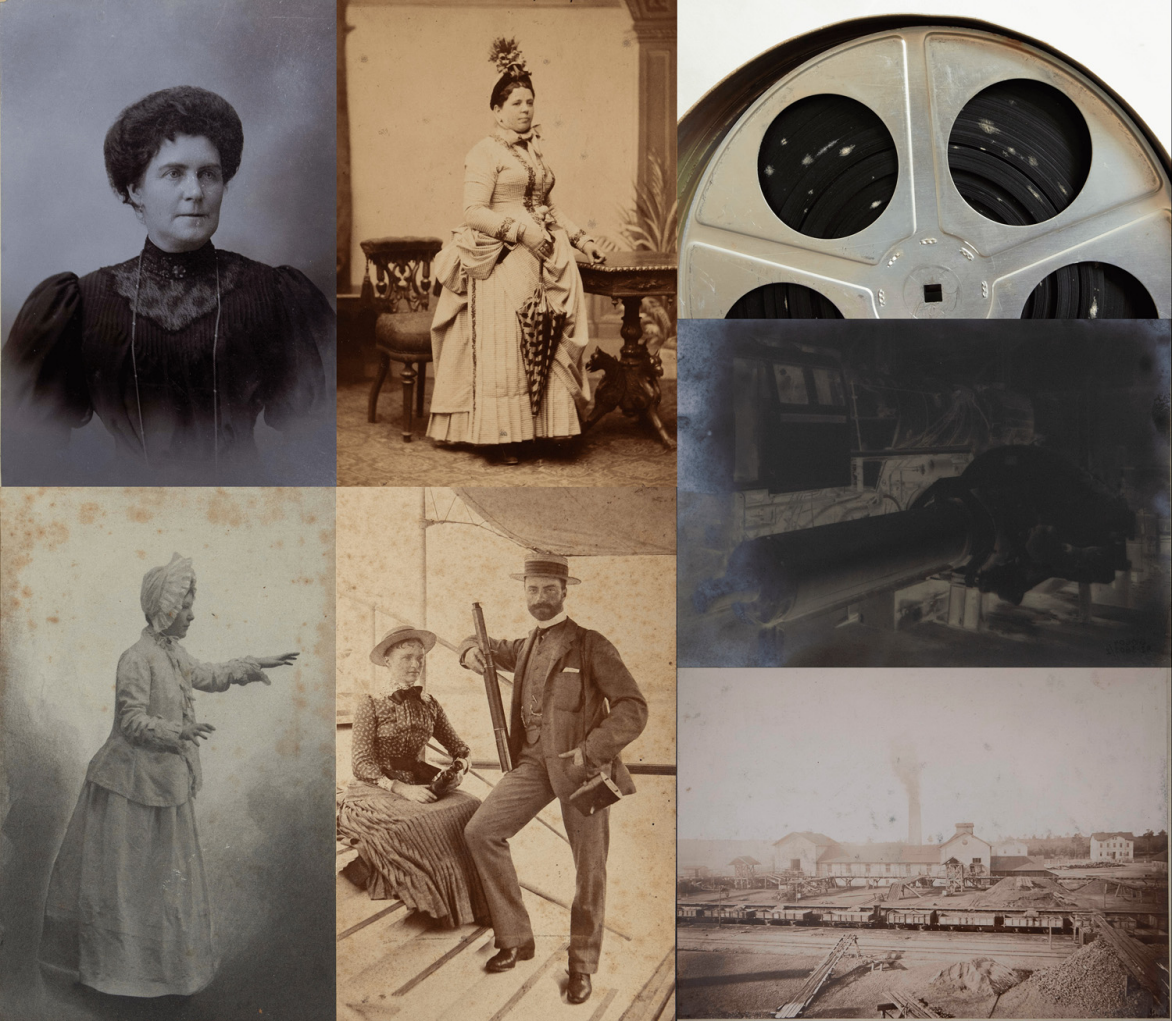
Selected audio-visual materials visibly colonized by microorganisms. From left: collodion photography, albumen photography, gelatine film, gelatine photography, albumen photography, gelatine negative photography, and albumen photography.

Like other cultural heritage objects, audio-visual materials (cinematographic film reels and positive and negative photographs) are often colonized by microorganisms, including fungi and bacteria (1, 2). These colonizing microorganisms can subsequently cause damage to materials through their growth and metabolic activity (3). One of the most common manifestations of damage for audio-visual materials is chromatic alteration, which causes colored stains on the materials (Fig. 1) (4). In addition to chromatic alterations, physical damage or even the total decomposition of the material may occur, mainly by microorganisms producing enzymes such as lipases, catalases, proteases, cellulases, or ligninases (5). Due to their higher moisture requirements, bacteria have a limited impact on the biodeterioration process of audio-visual materials compared to fungi (4). However, even bacteria have been shown to be abundant on cultural heritage objects and can cause not only material deterioration but also health problems for the employees (4, 6).

All photographic and cinematographic materials are composed of organic and inorganic components that microorganisms use for their growth and metabolism under appropriate conditions (7). The composition of audio-visual materials is highly varied and is usually composed of two layers, one of which is a carrier onto which a second light-sensitive layer (an emulsion of a light-sensitive substance and a binder) is deposited (8).

The options for monitoring microbial contamination, in general, can be divided into culture-dependent and culture-independent approaches. Although almost 20 years have passed since the commercial availability of next-generation sequencing, a culture-dependent approach still prevails in the field of cultural heritage (2). If we review existing studies focused on the contamination of audio-visual materials, at least half of them only address the identification of microscopic fungi. Some recent examples include studies by Borrego et al. (9), Branysova et al. (10), and Kwiatkowska et al. (11). Other studies looked at the identification of both domains, including Puskarova et al. (12), Sclocchi et al. (7), and Szulc et al. (6). In the last of the above-mentioned studies by Szulc et al. (6), in which Illumina MiSeq sequencing was used, the authors concluded that a high biodiversity of bacterial genera was detected on historical photographs, and some of these genera may be responsible for their biodegradation.

Although bacteria play an essential role in the biodeterioration of audio-visual materials, to the best of our knowledge, to date, no study has deeply focused on their occurrence. Previous studies mostly focused their attention exclusively on audio-visual materials with one particular binder type; namely, Puskarova et al. (12) studied albumen, and Sclocchi et al. (7) and Szulc et al. (6) studied gelatin. Moreover, these studies were restricted to a small number of samples (two to five photographs) without incorporating air analysis. Therefore, this work extends existing knowledge, compares the colonizing communities on various types of audio-visual materials (altogether 60 samples), and focuses on the link between air contamination and colonizing bacteria on audio-visual materials. For this purpose, we collected samples from four different archives in the Czech Republic. Our hypotheses were based on our previous publication that focused on monitoring fungi in archives in the Czech Republic (10). We postulated that the factor locality (indoor environmental conditions, including air contamination) would be the primary determinant of bacterial community composition and the key driver of variation in the data set.

## RESULTS

**FIG 2:**
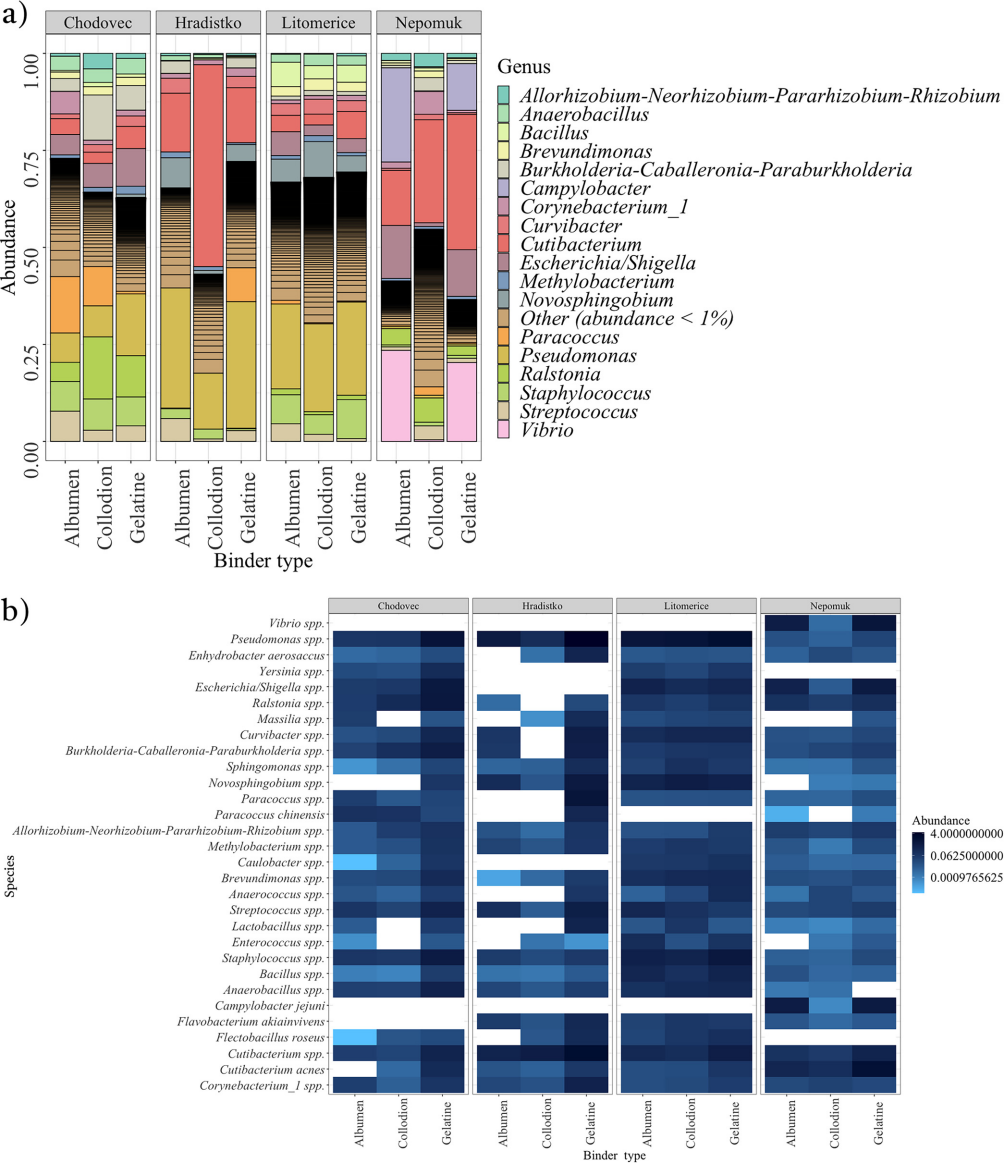
Distribution of bacteria on audio-visual materials with different binder types (albumen, collodion, and gelatine) at different localities (Chodovec, Hradistko, Litomerice, and Nepomuk). (a) Relative abundance of bacterial genera. (b) Heatmap of the 30 most abundant bacterial species.

**FIG 3:**
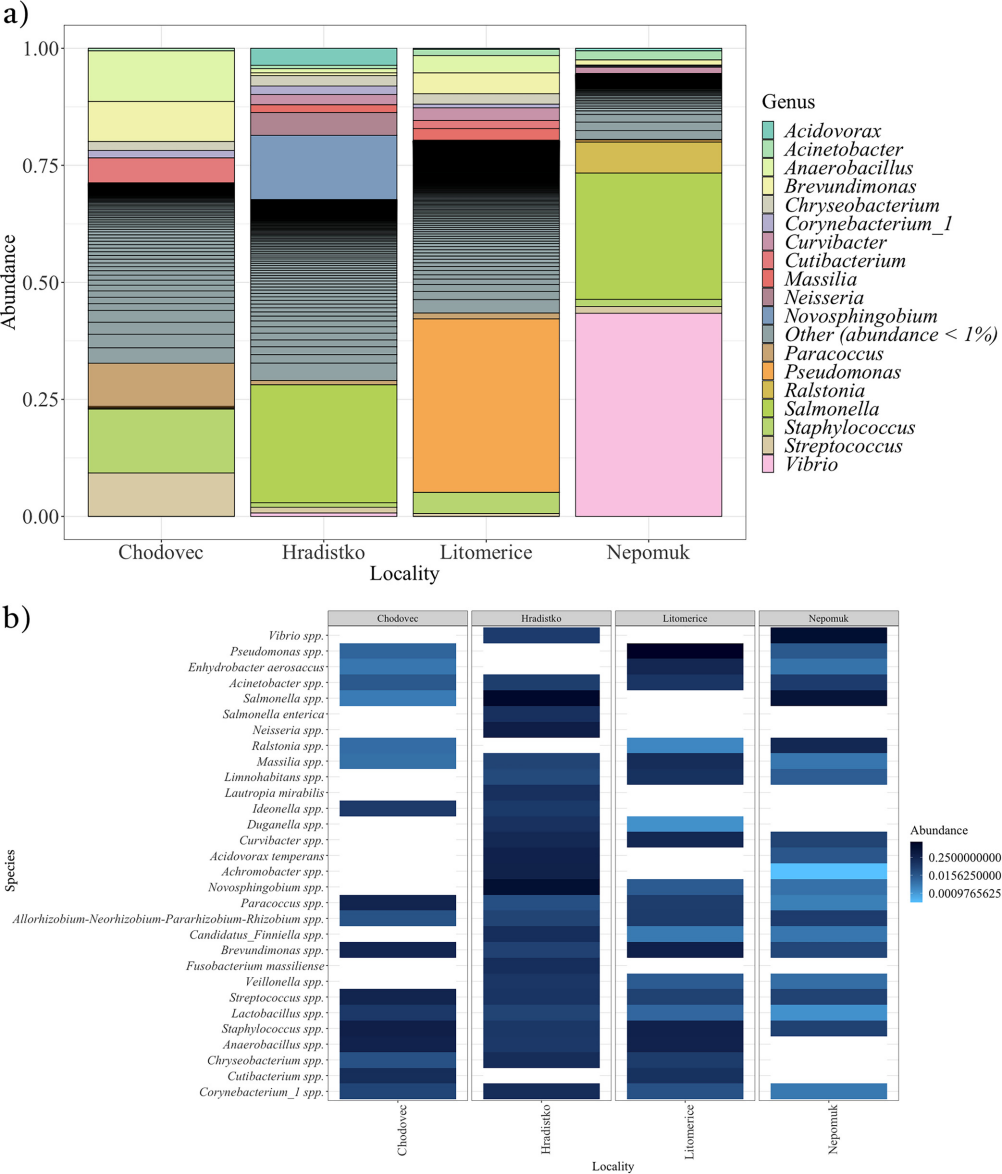
Distribution of bacteria in air at different localities (Chodovec, Hradistko, Litomerice, and Nepomuk). (a) Relative abundance of bacterial genera. (b) Heatmap of the 30 most abundant bacterial species.

**FIG 4:**
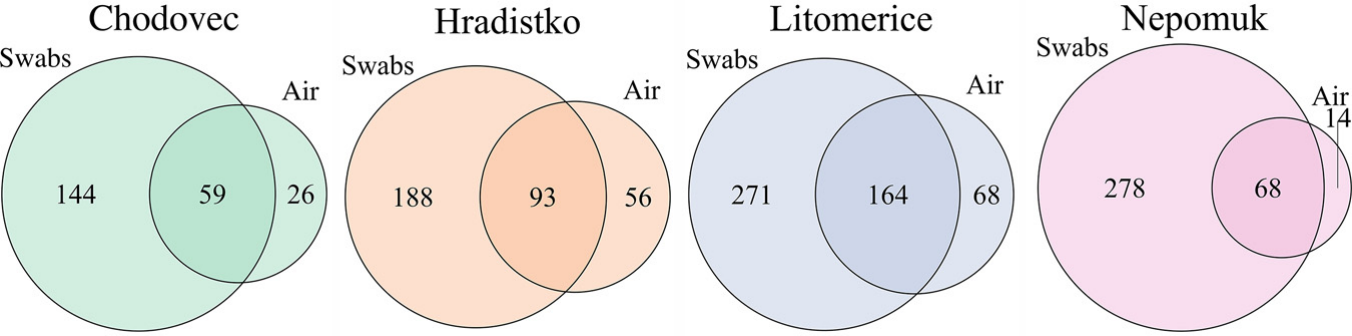
Intersections between bacterial genera on audio-visual materials (swabs) and in the air at different archives (Chodovec, Hradistko, Litomerice, and Nepomuk).

**FIG 5:**
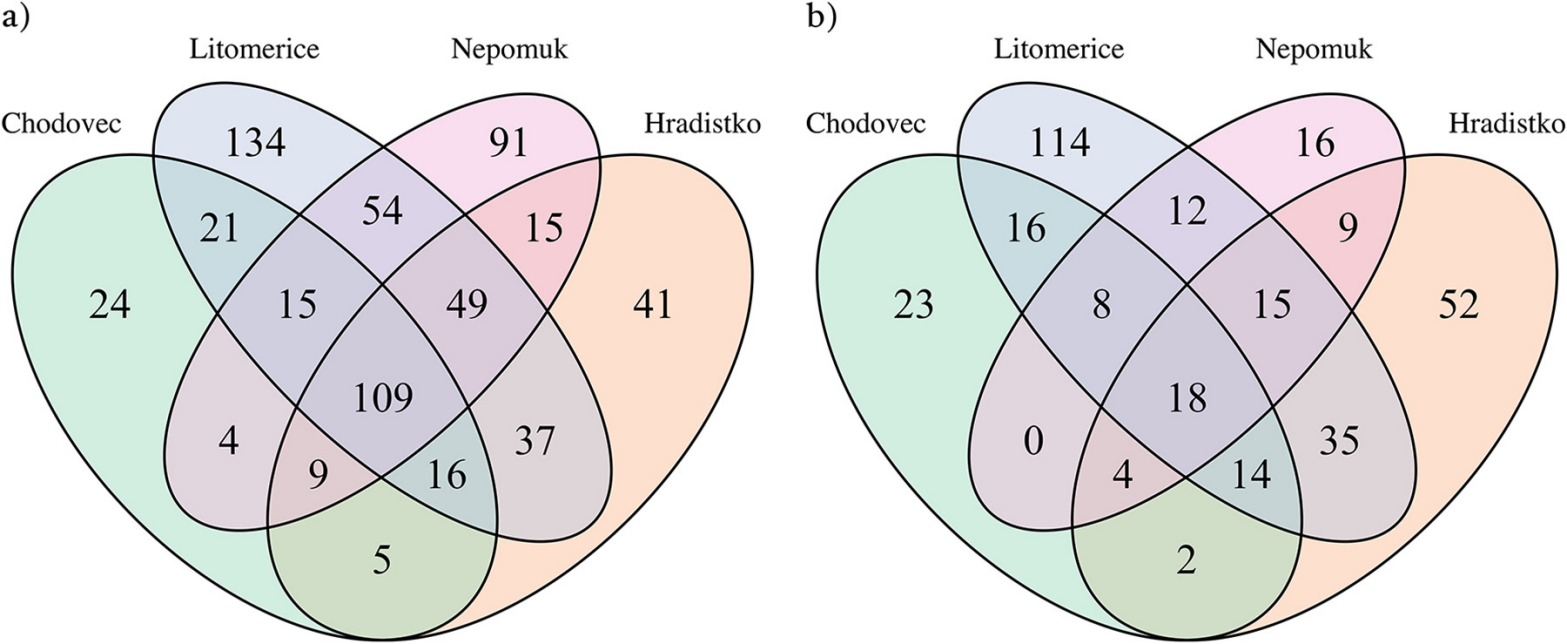
Intersections of bacterial genera on audio-visual materials (a) and in the air (b) at different localities (Chodovec, Hradistko, Litomerice, and Nepomuk).

This study involved the collection of a minimum of 2 air samples and 10 audio-visual samples from each archive, including samples with different binder types (albumen, gelatin, and collodion), resulting in a total of 13 air samples and 60 audio-visual samples analyzed (Tables 1 and 2). DNA was extracted from the samples and prepared into a DNA library for amplicon sequencing of the 16S rRNA gene using the Illumina MiSeq method. Results are presented in Fig. 2 to 5 and Tables 3 and 4.

**TABLE 1:** Audio-visual materials analyzed from archives in the Czech Republic

Locality	Sample no.	Type of audio-visual material	Binder	Carrier
State Regional Archive Prague-Chodovec	1S	Negative photography	Gelatine	Glass
	2S	Negative photography	Gelatine	Glass
	3S	Negative photography	Gelatine	Cellulose nitrate
	4S	Positive photography	Albumen	Paper
	5S	Positive photography	Collodion	Baryta paper
	6S	Positive photography	Gelatine	Baryta paper
	7S	Positive photography	Gelatine	Baryta paper
	13S	Film	Gelatine	Cellulose acetate
	14S	Film	Gelatine	Cellulose acetate
	15S	Film	Gelatine	Polyester
State District Archive Hradistko	1S	Positive photography	Albumen	Paper
	2S	Positive photography	Collodion	Baryta paper
	3S	Positive photography	Gelatine	Baryta paper
	4S	Positive photography	Albumen	Paper
	6S	Positive photography	Gelatine	Baryta paper
	7S	Positive photography	Gelatine	Baryta paper
	8S	Positive photography	Gelatine	Baryta paper
	9S	Positive photography	Gelatine	Baryta paper
	10S	Positive photography	Gelatine	Baryta paper
	11S	Positive photography	Gelatine	Baryta paper
	12S	Negative photography	Gelatine	Cellulose nitrate
	13S	Negative photography	Gelatine	Glass
	14S	Negative photography	Gelatine	Cellulose nitrate
	15S	Film	Gelatine	Cellulose acetate
	16S	Film	Gelatine	Cellulose acetate
	17S	Film	Gelatine	Cellulose acetate
	18S	Film	Gelatine	Cellulose acetate
	20S	Film	Gelatine	Cellulose acetate
	21S	Film	Gelatine	Polyester
	22S	Film	Gelatine	Cellulose acetate
State Regional Archive Litomerice	1S	Positive photography	Gelatine	Paper
	2S	Positive photography	Gelatine	Paper
	3S	Positive photography	Gelatine	Paper
	4S	Film	Gelatine	Cellulose acetate
	5S	Negative photography	Gelatine	Glass
	6S	Positive photography	Collodion	Paper
	7S	Negative photography	Gelatine	Cellulose acetate
	8S	Positive photography	Albumen	Paper
	9S	Positive photography	Collodion	Paper
	10S	Positive photography	Albumen	Paper
	12S	Positive photography	Collodion	Paper
	13S	Positive photography	Albumen	Paper
	14S	Positive photography	Collodion	Paper
	15S	Positive photography	Gelatine	Paper
	16S	Positive photography	Albumen	Paper
	17S	Positive photography	Albumen	Paper
	18S	Positive photography	Gelatine	Paper
	19S	Positive photography	Collodion	Paper
	21S	Positive photography	Collodion	Baryta paper
	22S	Positive photography	Gelatine	Paper
State District Archive Nepomuk	1S	Positive photography	Collodion	Baryta paper
	2S	Positive photography	Gelatine	Paper
	3S	Positive photography	Albumen	Paper
	4S	Positive photography	Albumen	Paper
	5S	Positive photography	Albumen	Paper
	6S	Positive photography	Gelatine	Paper
	7S	Negative photography	Gelatine	Glass
	8S	Negative photography	Gelatine	Cellulose nitrate
	9S	Negative photography	Gelatine	Glass
	10S	Film	Gelatine	Cellulose acetate

**TABLE 2:** Overview of storage conditions in the analyzed archives

Archive	Public access	Depository size	Air conditioning	Type of filter	Annual temp	Annual humidity	Type of furniture	Cleaning
Hradistko	Yes	198.1 to 564.5 m^3^	Yes	None	13.9°C	44.8 to 54.7%	Metal, mobile, immobile	1× per mo
Chodovec	Yes	580 m^3^	Yes	HEPA	18°C	45%	Metal, immobile	1 to 2× per yr
Litomerice	Yes	76 to 1,275 m^3^	No	None	19 to 21°C	44 to 55.5%	Metal, immobile	4× per yr
Nepomuk	Yes	97.5 to 1,950 m^3^	Yes	F5	17 to 18°C	41 to 57%	Metal, immobile	2× per yr/as required

**TABLE 3:** Influence of different factors on the composition of the bacterial community (PERMANOVA)

Factor	*R* ^2^	*P* value
Locality	0.285	0.001^*a*^
Type of carrier	0.060	0.647
Type of binder	0.027	0.294
Type of audio-visual material	0.012	0.617

a Significant *P* value.

**TABLE 4:** Overview of indicator genera for one or more localities

Locality	Phylum	Class	Order	Family	Genus
Litomerice	*Bacillota*	*Bacilli*	*Lactobacillales*	*Enterococcaceae*	*Enterococcus*
Nepomuk	*Bacteroidota*	*Bacteroidia*	*Flavobacteriales*	*Flavobacteriaceae*	*Myroides*
	*Campylobacterota*	*Campylobacteria*	*Campylobacterales*	*Campylobacteraceae*	Campylobacter
	*Pseudomonadota*	*Gammaproteobacteria*	*Enterobacteriales*	*Enterobacteriaceae*	*Atlantibacter*
	*Pseudomonadota*	*Gammaproteobacteria*	*Enterobacteriales*	*Enterobacteriaceae*	*Buttiauxella*
	*Pseudomonadota*	*Gammaproteobacteria*	*Enterobacteriales*	*Enterobacteriaceae*	*Rahnella*
	*Pseudomonadota*	*Gammaproteobacteria*	*Vibrionales*	*Vibrionaceae*	*Vibrio*
Hradistko + Litomerice	*Bacillota*	*Erysipelotrichia*	*Erysipelotrichales*	*Erysipelotrichaceae*	*Erysipelothrix*
	*Pseudomonadota*	*Alphaproteobacteria*	*Paracaedibacterales*	*Paracaedibacteraceae*	*Candidatus Finniella*
	*Pseudomonadota*	*Alphaproteobacteria*	*Sphingomonadales*	*Sphingomonadaceae*	*Novosphingobium*
Hradistko + Nepomuk	*Pseudomonadota*	*Gammaproteobacteria*	*Enterobacteriales*	*Enterobacteriaceae*	Salmonella
Chodovec + Litomerice	*Bacillota*	*Bacilli*	*Bacillales*	*Bacillaceae*	*Anaerobacillus*
	*Bacillota*	*Bacilli*	*Bacillales*	*Bacillaceae*	*Bacillus*
	*Bacillota*	*Bacilli*	*Bacillales*	*Staphylococcaceae*	Staphylococcus
	*Pseudomonadota*	*Alphaproteobacteria*	*Caulobacterales*	*Caulobacteraceae*	*Caulobacter*
	*Pseudomonadota*	*Alphaproteobacteria*	*Rhizobiales*	*Xanthobacteraceae*	*Afipia*
	*Pseudomonadota*	*Gammaproteobacteria*	*Enterobacteriales*	*Enterobacteriaceae*	*Yersinia*
Chodovec + Nepomuk	*Pseudomonadota*	*Gammaproteobacteria*	*Alteromonadales*	*Shewanellaceae*	*Shewanella*
	*Pseudomonadota*	*Gammaproteobacteria*	*Betaproteobacteriales*	*Burkholderiaceae*	*Ralstonia*
Litomerice + Nepomuk	*Pseudomonadota*	*Gammaproteobacteria*	*Betaproteobacteriales*	*Burkholderiaceae*	*Pelomonas*
Chodovec + Hradistko + Nepomuk	*Bacillota*	*Bacilli*	*Lactobacillales*	*Streptococcaceae*	*Lactococcus*
	*Pseudomonadota*	*Gammaproteobacteria*	*Betaproteobacteriales*	*Burkholderiaceae*	*Burkholderia-Caballeronia-Paraburkholderia*
Chodovec + Litomerice + Nepomuk	*Deinococcota*	*Deinococci*	*Thermales*	*Thermaceae*	*Thermus*
	*Pseudomonadota*	*Alphaproteobacteria*	*Caulobacterales*	*Caulobacteraceae*	*Brevundimonas*
	*Pseudomonadota*	*Gammaproteobacteria*	*Enterobacteriales*	*Enterobacteriaceae*	Escherichia/*Shigella*
	*Pseudomonadota*	*Gammaproteobacteria*	*Oceanospirillales*	*Halomonadaceae*	*Halomonas*

### Bacteria identified on audio-visual materials and factors influencing their presence.

Eighteen bacterial genera with a relative abundance greater than 1% were identified on audio-visual materials (Fig. 2a), with visible differences and similarities between the most abundant genera across different archives. The Nepomuk archive had the most distinct results, with the most abundant genera being Campylobacter, *Cutibacterium*, Escherichia*/Shigella*, and *Vibrio*. In the Chodovec, Hradistko, and Litomerice archives, the genus Pseudomonas was abundant, but other genera, such as *Novosphingobium*, Staphylococcus, and *Cutibacterium* (as in the Nepomuk archive), were also relatively abundant. Fig. 2b shows the 30 most abundant bacterial species on audio-visual materials across all archives, with 11 identified at all localities and on all binder types, including *Allorhizobium*-*Neorhizobium*-*Pararhizobium*-*Rhizobium* spp., *Anaerobacillus* spp., *Bacillus* spp., *Brevundimonas* spp., *Corynebacterium_1* spp., *Cutibacterium* spp., *Methylobacterium* spp., Pseudomonas spp., *Sphingomonas* spp., Staphylococcus spp., and Streptococcus spp.

Based on our prior research on microscopic fungi on audio-visual materials (10), we postulated that certain factors might also influence the composition of the bacterial communities on such materials. Our hypothesis was that the structure of bacterial communities would be significantly influenced by the locality in which archival materials were stored. This association can be attributed to variations in the environmental conditions maintained in each archive, such as using different air conditioner filters, which can promote the occurrence of distinct microbial species. This hypothesis was verified using the permutational multivariate analysis of variance (PERMANOVA) method, where the locality was shown to be significant at the 95% probability level (Table 3). Further, because audio-visual materials are composed of multiple layers, we explored whether any of these layers (binder [albumen, collodion, or gelatin], carrier [baryta paper, cellulose acetate, cellulose nitrate, glass, paper, or polyester], or the type of audio-visual material [film or positive or negative photography]) affect the structure of the bacterial communities on the specific type of audio-visual materials. However, none of these factors had an effect demonstrated at the 95% level. Furthermore, of all the factors tested, the locality appeared to explain most of the variability in the structure of the bacterial communities (Table 3).

Because all other factors, except locality, came out as nonsignificant, a pairwise PERMANOVA was used to examine each pair of variables within each factor. At the 95% probability level, no pair was found to be significant. However, at the 90% level, the pairs positive photography versus film (adjusted *P* value [*P*_adj_] = 0.088) and collodion versus gelatin (*P*_adj_ = 0.076) were significant.

### Bacteria identified in archive air.

Given that locality determined by indoor environmental conditions, including microbial contamination of the air, was found to have the most significant effect, our attention was directed toward identifying the bacterial species present in the air. Eighteen bacterial genera with a relative abundance of greater than 1% were identified in the air (Fig. 3a). Notably, the most abundant genera varied between archives. Specifically, the genera Staphylococcus and *Anaerobacillus* were dominant in the Chodovec archive, while the genus Salmonella was clearly dominant in the Hradistko archive, the genus Pseudomonas was dominant in the Litomerice archive, and the genera *Vibrio* and Salmonella were dominant in the Nepomuk archive. A heatmap was also created for the 30 most abundant species in the air at the different localities (Fig. 3b), revealing that only 9 species were identified in all analyzed archives: Acinetobacter spp., *Allorhizobium*-*Neorhizobium*-*Pararhizobium*-*Rhizobium* spp., *Brevundimonas* spp., *Corynebacterium_1* spp., *Lactobacillus* spp., *Massilia* spp., *Paracoccus* spp., Staphylococcus spp., and Streptococcus spp.

As several abundant genera were identified in the air that matched those identified on the audio-visual materials (Fig. 2a and b and 3a and b), intersections were constructed between bacterial genera identified from audio-visual swabs and the air for each locality (Fig. 4). In all archives, it was found that more than 60% of the bacterial genera identified in the air of the archives were also identified on audio-visual materials. In the Nepomuk archive, this was over 82%. A significantly greater degree of overlap was observed for bacteria than for fungi in our prior investigation (10) in which the highest degree of overlap was recorded in Hradistko with only 38%.

### Comparison of identified bacteria across archives.

The distribution of the identified genera on audio-visual materials and in the air according to different localities is shown in Fig. 5a and b, respectively. Of the 624 bacterial genera identified on the audio-visual materials, 109 genera (17.5%) were identical for all localities. For the air, 18 genera (5.3%) were identified from a total of 338 genera across all localities. Comparing these two intersections, we found that all 18 genera identified in the air were also identified on audio-visual materials. These genera included Acinetobacter, *Allorhizobium*-*Neorhizobium*-*Pararhizobium*-*Rhizobium*, *Brevundimonas*, *Chryseobacterium*, *Corynebacterium_1*, *Flavobacterium*, *Fusobacterium*, *Lactobacillus*, *Marmoricola*, *Massilia*, *Methylobacterium*, *Micrococcus*, *Nocardioides*, *Paracoccus*, *Sphingomonas*, Staphylococcus, *Stenotrophomonas*, and Streptococcus.

Furthermore, an analysis of indicator microorganisms was performed. The results for one or more localities are shown in Table 4. Twenty-six indicator genera were obtained (*P*_adj_ < 0.05). No indicator genera were found for the Chodovec and Hradistko archives, while one genus was found for the Litomerice archive and as many as six genera were found for the Nepomuk archive.

## DISCUSSION

This study used a culture-independent approach based on DNA isolation to analyze microbial contamination on audio-visual materials and in the air of archives. DNA amplification and subsequent amplicon sequencing using the Illumina MiSeq method enabled us to obtain a comprehensive overview of the bacterial communities present on the materials and in the air of the archive depositories. Eighteen bacterial genera were identified on audio-visual material, and 18 genera were identified in the air, each with a relative abundance of greater than 1%. Some of these genera have also been identified in other studies examining the contamination of audio-visual materials.

Two previous studies have, like us, exclusively used culture-independent methods to examine bacterial communities on audio-visual materials. The first study by Buckova et al. (13) analyzed microbial contamination on cellulose-nitrate film and a gelatin-silver print by PCR, clone libraries, and sequencing. *Lactococcus* sp., Streptococcus sp., Pseudomonas sp., Pseudomonas cedrina, Clostridium straminisolvens, Pseudomonas gessardii, and Escherichia coli were identified on the film, while the photograph was found to contain Pseudomonas cedrina, Clostridium straminisolvens, Shigella sonnei, and Pseudomonas poae. Our results show some similarities, with all genera (apart from *Clostridium* and *Lactococcus*) being among the most abundant on the materials in our study. Of particular relevance is the genus Pseudomonas, which has been shown to play a role in the biodeterioration of audio-visual materials, has cellulolytic and proteolytic properties, and is considered a “gelatin liquefier” (14, 15).

A second study by Szulc et al. (6) used Illumina MiSeq sequencing and focused on gelatin photographs. The authors found a high diversity of bacterial genera, with *Mesorhizobium* and *Ralstonia* being among the most abundant. Other identified genera included *Burkholderia*, *Delftia*, *Enhydrobacter*, Escherichia-*Shigella*, *Paenibacillus*, *Saccharopolyspora*, and *Olsenella*. Our study also identified *Ralstonia* as well as the genera *Burkholderia* and Escherichia/*Shigella*. Of these, *Burkholderia* is notable in the context of audio-visual materials due to its association with biodeterioration and classification as a “gelatin liquefier” (14, 15). Additionally, Enhydrobacter aerosaccus, a representative of the genus *Enhydrobacter*, was among the 30 most abundant species on audio-visual materials. This genus is also linked to the biodeterioration of audio-visual materials (15).

Several studies have used either a culture-dependent approach or a combination of both methods for bacterial identification. Across these studies, the genera *Bacillus*, *Clostridium*, *Kocuria*, and Staphylococcus are frequently mentioned (4, 7, 12, 16–18). Our study also detected a high abundance of *Bacillus* and Staphylococcus. These two genera have been linked to the deterioration of various cultural artifacts, such as textiles, wall paintings, canvases, wood, paper, and parchment (15). Furthermore, these genera have demonstrated proteolytic activity, which presents a threat especially to audio-visual materials with a gelatinous binder. Additionally, members of the *Bacillus* genus have cellulolytic properties that pose a risk to collodion audio-visual materials (as the main component of collodion is cellulose nitrate) as well as the ability to produce alkaline serine proteases that are harmful to albumin materials (2).

The issue of microbial air contamination often goes unnoticed in studies, but it plays a crucial role in the contamination of materials. To fully address this problem, the inclusion of air analysis in research is essential. Our previous study (10) demonstrated the importance of air analysis in understanding the microbial contamination of audio-visual materials. This study confirms this by determining that the composition of bacterial communities on audio-visual materials is significantly affected by the conditions in the archive in which they are stored. Each archive has its own unique environmental conditions, such as temperature, humidity, and airflow, and it is crucial to take these factors into account in research. While some factors were not statistically significant at the 95% probability level, our results did reveal differences in bacterial community composition between certain individual factor levels, specifically collodion and gelatin as well as positive photography and film reels.

The observed differences in bacterial community composition between collodion and gelatin are likely to be attributed to their distinct chemical compositions. Collodion, which contains cellulose nitrate as its main component (19), is likely colonized by microorganisms with cellulolytic properties, while gelatin, a biopolymer derived from collagen denaturation (20), is likely targeted by microorganisms with proteolytic properties. Similarly, differences in bacterial community composition between positive photography and film reels may also be linked to their respective compositions. All the analyzed film reels had gelatin as a binder and cellulose acetate or polyester as a carrier. By contrast, the examined positive photographs incorporated a variety of binders, such as gelatin, albumin, and collodion, as well as paper and baryta paper (Table 1), leading to a more diverse range of bacterial species.

While microbial air contamination is often overlooked in the context of cultural heritage objects, a few studies have included it in their research. For example, Borrego et al. (4), Borrego et al. (18), and Guiamet et al. (17) monitored bacterial contamination in depositories storing audio-visual materials by using a sedimentation method and a culture-dependent approach. All of them identified the genus *Bacillus*, with the first- and last-mentioned studies also identifying other genera, such as Staphylococcus, Streptococcus, and *Serratia*. In our study, the genera Staphylococcus and Streptococcus were found to be among those with abundances higher than 1%. As these genera are commonly found on human and animal skin and are ubiquitous due to their adaptability, it is natural that they have already been identified in several archives (4, 21, 22). However, these genera can pose a threat to audio-visual materials due to their proteolytic properties (2).

Among the bacterial genera identified on audio-visual materials and in the air, the genera *Brevundimonas*, *Corynebacterium_1*, *Paracoccus*, Staphylococcus, and Streptococcus are worth mentioning, as their abundances were higher than 1%. While Staphylococcus and Streptococcus were previously mentioned, *Brevundimonas* is a genus that is widespread in the environment (23), and some of its members have demonstrated proteolytic activity and have been identified on objects subject to biodeterioration, such as Leonardo da Vinci’s Codex Atlantic pages (24) and waterlogged archaeological wood (25). For the remaining genera, members of *Corynebacterium* and *Paracoccus* have been associated with the biodeterioration of textiles and paintings on canvas (15). The genus *Corynebacterium* has been shown to produce color pigments (14), while the *Paracoccus* genus is a versatile organism that can use a wide range of organic compounds as carbon and energy sources (26) and is thus likely involved in the biodeterioration of audio-visual materials. In addition to the aforementioned information, it is noteworthy to mention that the genus *Corynebacterium* is classified under the *Actinobacteria* phylum, which is recognized for its capability to degrade cultural heritage objects (27). Among the genera belonging to this phylum, our study identified another genus, *Cutibacterium*, which exhibited an abundance of higher than 1%. We are aware that underestimation of the presence of the phylum *Actinobacteria* can be attributed to the choice of primers. The findings of Abellan-Schneyder et al. (28) support this notion, as they demonstrate that the detection of the *Actinobacteria* phylum as well as other bacterial phyla, such as *Tenericutes*, *Lentisphaerae*, and *Verrucomicrobia*, can vary when different primer pairs are used. Additionally, a study by Varliero et al. (29) revealed different relative abundances of the *Actinobacteria* phylum across various primer sets, with a smaller percentage of representation observed when primers targeting the V4-V5 region of the 16S rRNA gene, which was also targeted by our primers, were compared to primers targeting the V8-V9 region. However, targeting the V4-V5 region of 16S rRNA in our study was chosen because it is one of the most commonly targeted regions in short amplicon sequencing (28).

In light of the significant intersections (more than 60% for each archive) between the genera identified on audio-visual materials and in the air, it is confirmed that air is one of the main sources of microbial contamination in archives. Consequently, it is essential to undertake measures aimed at maintaining clean air within these archival environments. The minimum should be to use high-quality filters in air conditioning systems (for instance, HEPA filters) and ensure their regular replacement to minimize the entry of microorganisms. Unfortunately, relying solely on filters in the air conditioning system is inadequate to address microbial contamination in archives. This insufficiency is evident in the Chodovec archive, where HEPA filters are used; however, there is still a substantial overlap of bacterial genera, with more than 69% identified in the air also found on audio-visual materials. Consequently, we recommend the incorporation of alternative cleaning methods, such as UV light cleaning or photocatalytic or plasma cleaning methods (30). The limited use of air conditioning filters, if any, in heritage buildings can likely be attributed to cost constraints (typically state-owned enterprises) as well as potential installation challenges arising from the older infrastructure. Therefore, future research in the cultural heritage sector should focus on exploring and developing innovative approaches to identify affordable and easily installable air disinfection equipment. Implementing such measures would undoubtedly lead to a significant reduction in the presence of microorganisms in the air, thereby ensuring the safety of cultural heritage objects and the staff working in these institutions.

Analysis of indicator genera can help in recognizing genera that are significantly linked to specific localities (31). Several of these indicator genera were found to have an abundance of greater than 1% in both air and audio-visual material samples. For instance, the genus *Vibrio* was significantly linked to the Nepomuk archive. Some species of the genus *Vibrio* are known to possess cellulolytic activity (32) or produce alkaline serine proteases (33), which could potentially pose a threat to collodion and albumin audio-visual materials, respectively (2). Other significant genera included *Anaerobacillus*, *Brevundimonas*, *Novosphingobium*, *Ralstonia*, and Staphylococcus. While the roles of the genera *Brevundimonas* and Staphylococcus in the biodeterioration of audio-visual materials have been discussed previously, the remaining genera warrant further comment. Cellulolytic activity has been documented for the genus *Novosphingobium*, and certain species are capable of efficiently degrading lignocellulosic waste (34). Conversely, the genus *Anaerobacillus* has been shown to degrade gelatin (35), and the genus *Ralstonia*, which was also among the most abundant genera in the study by Szulc et al. (6), has been found to form brown pigments. These bacteria have also been found to produce enzymes that degrade chemicals used in paper production (36). Thus, for audio-visual materials with a paper carrier, the genus *Ralstonia* may contribute to the biodeterioration process.

Several bacterial genera, which have been identified as more abundant and potentially hazardous to audio-visual materials, were the subject of discussion above. However, the metabolic activity of bacteria is generally limited under normal storage conditions. In our archives, this limitation was mainly attributed to relatively lower relative humidity (approximately 50%) rather than moderate temperatures (with one exception around 20°C). Nevertheless, it is imperative to acknowledge the impact of temperature and humidity fluctuations, which significantly contribute to the deterioration process and prove arduous to mitigate effectively. These fluctuations can create optimal conditions for the metabolic activity of harmful bacteria multiple times throughout the year. Furthermore, with the emergence of the energy crisis, transient fluctuations in temperature and humidity may occur more frequently as energy-saving measures are implemented and archival facilities reduce their usage of air conditioning.

### Conclusion.

This study investigated the bacterial diversity on audio-visual materials and in the air of Czech Republic archives using a noninvasive, culture-independent approach. The results revealed a wide range of bacterial genera, some of which may pose a potential threat to audio-visual materials due to their ability to degrade some of the components of photographs and film reels. Therefore, work done to prevent the contamination of or disinfect already-infested materials should target these genera. Moreover, this study highlights the importance of microbial air screening, as a clear correlation was found between bacterial genera on audio-visual materials and in the air of depositories. More than 60% of the species identified in the air of each archive were also found on audio-visual materials, and the PERMANOVA statistical analysis confirmed that locality significantly affects the composition of the bacterial communities. However, carrier type, binder type, and audio-visual material type did not have a significant effect on bacterial communities. Overall, our findings emphasize the need for continued research in this area to identify potential threats to audio-visual materials and to develop strategies to mitigate the risk of biodeterioration in archives.

## MATERIALS AND METHODS

### Examined materials and archive description.

The audio-visual material samples were taken from four archives: the state regional archives Litomerice and Prague-Chodovec and the state district archives Nepomuk and Hradistko. In each archive, swabs of audio-visual materials with different binders (gelatin, albumen, and collodion) and different carriers were collected. Table 1 provides an overview of all analyzed audio-visual materials. Some of the analyzed audio-visual materials are shown in Fig. 1. In addition to swabbing, air sampling was also conducted in each archive. The characteristics of the archival environments are provided in Table 2.

### Sampling.

Preparation for sampling and the sampling process were the same as described in our previous study (10). Before sampling, instruments such as tweezers, scalpels, and scissors were exposed to gamma radiation for sterilization. Similarly, 0.8% saline solution from water for molecular microbiology was also exposed to gamma radiation. Polytetrafluoroethylene (PTFE) membranes with a porosity of 0.22 μm and a diameter of 90 mm (Merck Millipore, Germany) were sterilized using an autoclave and subsequently illuminated with a UV lamp.

Proportional swabbing of each audio-visual material was carried out to ensure consistency in the sampling approach. The method for sampling the materials was chosen to be as gentle to the material as possible. Thus, sterile polyurethane sponges (World Bio-Products, Woodinville, WA, USA) were used. Air sampling was conducted at a height of approximately 1 m within the shelves, precisely where the audio-visual materials were stored. The sampling locations varied, ranging from the central area of the room to shelves positioned near the wall. An active intake of 3,000 liters of air onto PTFE Fluoropore membranes using a MAS-100 Eco aeroscope (Merck Millipore, Germany) was used for air sampling. The swabs and the PTFE membranes were stored at −20°C for further analysis.

### Metagenomic DNA isolation.

Isolation of metagenomic DNA was performed the same as we described in our previous study (10). Briefly, 30 mL of gamma-sterilized saline solution was added to the swab sponges, and the samples were homogenized using a stomacher for 3 min. These extracts were filtered through filtration cups with a polyethersulfone (PES) membrane (porosity of 0.2 μm and diameter of 50 mm; VWR International, Czech Republic). Next, the PES membranes were cut out and put into PowerWater DNA bead tubes from a DNeasy PowerWater kit (Qiagen, Germany). The next procedure followed the instructions from the commercial kit with a modification in the last step, in which DNA elution was performed using 70 μL of nuclease-free water. The PTFE membranes from the aeroscope were sterile quartered. Two-quarters of each membrane were processed in the same manner as the PES membranes.

### Amplicon preparation and sequencing.

Amplicons of the 16S rRNA gene were prepared by two-step PCR. The specific primers forward 515F-BAF 5-GTGYCAGCMGCNGCGG-3 and reverse 926R-BAF 5-CCGYCAATTYMTTTRAGTTT-3 (37) were used for amplification of the V4-V5 region (all Sigma-Aldrich, St. Louis, MO, USA). The composition of the master mix and temperature programs were taken from a study by Kracmarova et al. (37) with a few modifications. For the first PCR, the total reaction volume of 15 μL contained nuclease-free water, 1 μM each of the appropriate primers, 20 mU/μL KAPA HiFi HotStart ReadyMix (KAPA Biosystems, Wilmington, MA, USA), and 2 μL of template. The temperature program for the first PCR was as follows: 5 min of denaturation at 95°C, followed by 30 cycles of 20 s at 98°C, 15 s at 56°C, and 15 s at 72°C. The final extension was run at 72°C for 5 min. Each sample was made in 8 replicates, and after PCR, the corresponding replicates were merged and concentrated using a Genomic DNA Clean & Concentrator kit (ZYMO Research, Irvine, CA, USA).

For the second PCR, also called the index PCR, the total reaction volume of 25 μL contained nuclease-free water, 1 μM each of the appropriate primers, 20 mU/μL KAPA HiFi HotStart ReadyMix (KAPA Biosystems, Wilmington, MA, USA), and 1 μL of template. The temperature program for the index PCR was as follows: 5 min of denaturation at 95°C, followed by 13 cycles of 20 s at 98°C, 15 s at 50°C, and 15 s at 72°C. The final extension was run at 72°C for 5 min.

The success of each PCR was verified by 1.5% (wt/vol) agarose gel electrophoresis (120 V for 60 min). The prepared and verified amplicons were sent to the University of Fairbanks, AK, to be processed with an Illumina MiSeq high-throughput sequencing platform.

### Data processing and multivariate statistical analysis.

Taxonomy was assigned to individual sequences using the R programming language (38) and its DADA2 package (39), according to the DADA2 pipeline, version 1.16. First, forward and reverse primers were removed from the sequences. Sequences were then sorted according to the following parameters: truncLen = c(227, 175), maxN = 0, maxEE = (2, 2), and truncQ = 2. All chimeric sequences were then found and removed using the “consensus” method. Sequences that differed in only one base were combined, and the more abundant sequence was kept to avoid potential errors. The taxonomy itself was assigned to individual amplicon sequence variants (ASVs) using the database for the 16S rRNA gene silva_nr_v132_train_set.fa.gz (40).

Before starting any statistical analyses, the appropriate negative controls were subtracted from the samples in the R programming language, and the data were normalized using the “compositional” method. Subsequent multivariate statistical analysis was performed using the vegan (41) and phyloseq (42) packages of the R programming language. Stacked bar graphs were used to display the relative abundances of bacterial genera on audio-visual materials with different binder types and in the archive air. Heatmaps were then made to show the 30 most abundant species on the audio-visual materials and in the air. Venn diagrams were used to show the distribution of identified bacterial genera between localities (on audio-visual materials and in the air separately) and within localities by sample type (swabs versus air samples). Indicator genera for each locality were determined using an analysis inspired by indicator species analysis (the indicspecies package) (43), and false discovery rate (FDR) was used to calculate adjusted *P* values. Next, the data set was converted using the Hellinger transformation, and the statistical significance of several factors (location, archival type, substrate type, and binder type) was determined using permutational multivariate analysis of variance (PERMANOVA) based on Bray-Curtis distance. Furthermore, a pairwise PERMANOVA was performed to test for significant differences between pairs within the factors of interest. Again, FDR was used to calculate adjusted *P* values, and permutations did not include mixing samples from different localities.

### Data availability.

Raw data were uploaded to the NCBI Sequence Read Archive (SRA) under BioProject accession number PRJNA941910.
